# Dissection of the Role of VIMP in Endoplasmic Reticulum-Associated Degradation of CFTRΔF508

**DOI:** 10.1038/s41598-018-23284-8

**Published:** 2018-03-19

**Authors:** Xia Hou, Hongguang Wei, Carthic Rajagopalan, Hong Jiang, Qingtian Wu, Khalequz Zaman, Youming Xie, Fei Sun

**Affiliations:** 1 Department of Physiology, Wayne State University School of Medicine, Detroit, MI 48201 USA; 2 Department of Biochemistry and Molecular Biology, Jiamusi University School of Basic Medicine, Jiamusi, Heilongjiang 154007 China; 3 Department of Pediatrics, Case Western Reserve University, Cleveland, OH 44106 USA; 4 Karmanos Cancer Institute and Department of Oncology, Wayne State University School of Medicine, Detroit, MI 48201 USA

## Abstract

Endoplasmic reticulum (ER)-associated protein degradation (ERAD) is an important quality control mechanism that eliminates misfolded proteins from the ER. The Derlin-1/VCP/VIMP protein complex plays an essential role in ERAD. Although the roles of Derlin-1 and VCP are relatively clear, the functional activity of VIMP in ERAD remains to be understood. Here we investigate the role of VIMP in the degradation of CFTRΔF508, a cystic fibrosis transmembrane conductance regulator (CFTR) mutant known to be a substrate of ERAD. Overexpression of VIMP markedly enhances the degradation of CFTRΔF508, whereas knockdown of VIMP increases its half-life. We demonstrate that VIMP is associated with CFTRΔF508 and the RNF5 E3 ubiquitin ligase (also known as RMA1). Thus, VIMP not only forms a complex with Derlin-1 and VCP, but may also participate in recruiting substrates and E3 ubiquitin ligases. We further show that blocking CFTRΔF508 degradation by knockdown of VIMP substantially augments the effect of VX809, a drug that allows a fraction of CFTRΔF508 to fold properly and mobilize from ER to cell surface for normal functioning. This study provides insight into the role of VIMP in ERAD and presents a potential target for the treatment of cystic fibrosis patients carrying the CFTRΔF508 mutation.

## Introduction

Elimination of misfolded endoplasmic reticulum (ER) proteins by the ER-associated protein degradation (ERAD) pathway is an important physiological adaptation to ER stress^1,2^. The process of ERAD can be divided into three major steps: recognition of misfolded or mutated proteins in the ER, retro-translocation of ER proteins into the cytosol, and ubiquitin-dependent degradation by the proteasome. These steps are integrated through the actions of multi-subunit protein machineries in the ER region. One of the key components of the ERAD system is the Derlin-1/VCP/VIMP complex^3–5^. Derlin-1, a homologue of yeast Der1, associates with various substrates as they move across the ER membrane. VCP (valosin-containing protein, also known as p97) is a homologue of the yeast Cdc48 ATPase. Assisted by a host of cofactors such as Ufd1-Npl4, p47, and UBXD1, VCP recognizes and processes ubiquitylated substrates^6–9^. The ATP-driven unfolding activity of VCP provides the force to extract protein substrates out of the ER membrane. VIMP (also named SelS for Selenoprotein S) is a 189-amino acid small protein, which was initially identified as a member of the selenoprotein family characterized by carrying a selenocysteine (Sec, U, Se-Cys) amino acid^10^. Subsequent studies showed that VIMP is located at the ER membrane and interacts with both Derlin-1 and VCP, implying that VIMP participates in ERAD by serving as a linker between Derlin-1 and VCP^3–5^. Recent studies suggest that VIMP may be implicated in several proteostasis-related diseases, including Hashimoto’s thyroiditis, inflammation, fertility, and Alzheimer’s disease^11–14^.

Cystic fibrosis (CF) is one of the most common genetic diseases in the USA. CF is caused by mutations of the cystic fibrosis transmembrane conductance regulator (CFTR) gene^15^. The most common and prominent CF mutation is the deletion of a phenylalanine residue at position 508, resulting in a mutant protein CFTRΔF508, which is misfolded and degraded by the ERAD system during early biogenesis. This results in almost no membrane expression of CFTR and no chloride channel function^16^. Yet, CFTRΔF508, once escaping from ERAD, can migrate to the plasma membrane and retain substantial CFTR chloride channel function. Therefore, understanding the mechanism underlying CFTRΔF508 degradation by ERAD may provide crucial information for the development of drugs to treat CF patients. Our previous studies indicated that Derlin-1 and VCP are required for efficient degradation of CFTR, especially CFTRΔF508^17^. Here we investigate the role of VIMP in the degradation of CFTRΔF508. We demonstrate that VIMP interacts with CFTRΔF508 and both co-localize in the ER region. Furthermore, VIMP binds the RNF5 ubiquitin E3 ligase. Over-expression of VIMP reduces the steady-state level of CFTRΔF508 by shortening its half-life. In contrast, silence of VIMP expression increases the stability of CFTRΔF508. Interestingly, blocking early degradation of CFTRΔF508 by silence of VIMP significantly enhances the rescue effect of CF corrector VX809. This study reveals the role of VIMP in ERAD of CFTRΔF508 and presents a potential target for the treatment of CF patients.

## Results

### **VIMP is associated with CFTR**Δ**F508 in human airway cells**

**Figure 1:**
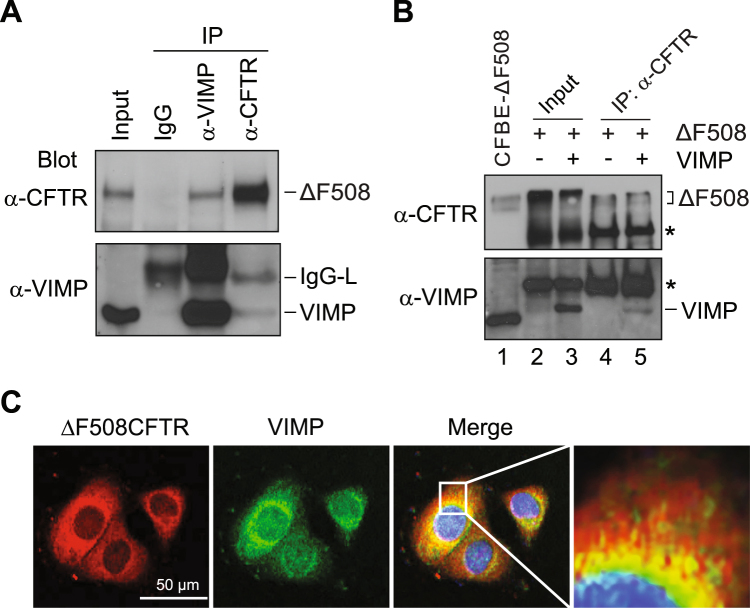
Association of VIMP with CFTRΔF508 in human airway epithelia cells. (**A**) Co-IP of VIMP with CFTRΔF508. Cell extracts prepared from CFBE41o^−^-CFTRΔF508 cells were subjected to IP with pre-immune IgG (lane 2), anti-VIMP (lane 3), and anti-CFTR (lane 4) antibodies, followed by immunoblotting with anti-CFTR (upper panel) and anti-VIMP (lower panel) antibodies. (**B**) Co-IP of VIMP and CFTRΔF508 produced by the TNT T7 coupled reticulocyte lysate system. The TNT T7 coupled reticulocyte lysates containing DNA templates for CFTRΔF508 alone (lanes 2, 4) or both CFTRΔF508 and VIMP (lanes 3, 5) were subjected to IP with an anti-CFTR antibody (lanes 4, 5), followed by immunoblotting with anti-CFTR and anti-VIMP antibodies, respectively. The same lysates were also applied to simple Western blotting as input control (lanes 2, 3). Cell lysate of CFBE-ΔF508 cells was used as a control to indicate the positions of ΔF508CFTR and VIMP on the blots (lane 1). Asterisks mark proteins in the TNT T7 coupled reticulocyte lysate cross-reactive with anti-CFTR and anti-VIMP antibodies. (**C**) Colocalization of VIMP with CFTRΔF508 at ER membrane dilations. CFBE41o^−^-CFTRΔF508 cells were incubated with anti-CFTR and anti-VIMP primary antibodies, followed by staining with Alexa 568 (red)-labeled secondary antibody for CFTR and Alexa 488 (green)-labeled secondary antibody for VIMP. The images were generated by confocal microscopy.

To determine the involvement of VIMP in ERAD of CFTRΔF508, we examined if VIMP interacts with CFTRΔF508 by co-immunoprecipitation (IP)/immunoblotting analysis. A CFTRΔF508 expressing cell line was established from CFBE41o^−^ (CFBE), a well-characterized human airway epithelial cell line. CFBE was derived from the bronchial epithelial cells of a CF patient with cftr∆F508/∆F508 genetic background and with no detectable expression of the mutant protein^18^. We found that CFTRΔF508 was co-immunoprecipitated with VIMP by an anti-VIMP antibody but not by a pre-immune normal IgG (Fig. 1A, lanes 2, 3). The association of CFTRΔF508 with VIMP in CFBE41o^−^-CFTRΔF508 cells was also confirmed by reciprocal co-IP/immunoblotting (Fig. 1A, lane 4). The technical difficulty of purifying CFTRΔF508 transmembrane protein from bacterial cells prevented us from definitely testing if VIMP directly binds CFTRΔF508 using *in vitro* binding analysis. To address this problem, we applied the TNT T7 coupled reticulocyte lysate system to produce full-length CFTRΔF508 and VIMP proteins. Co-IP experiments indicated that CFTRΔF508 and VIMP indeed formed a complex (Fig. 1B). This result suggests that these two proteins likely bind to each other. To further demonstrate the interaction between CFTRΔF508 and VIMP, we examined the subcellular location of CFTRΔF508 and VIMP in CFBE41o^−^-CFTRΔF508 cells using immunofluorescence microscopy. As shown in Fig. 1C, CFTRΔF508 and VIMP were co-localized throughout the ER network, mainly to the perinuclear region at ER membrane dilations. Together, these results indicate that VIMP physically binds to CFTRΔF508.

### VIMP facilitates proteasomal degradation of CFTRΔF508

**Figure 2:**
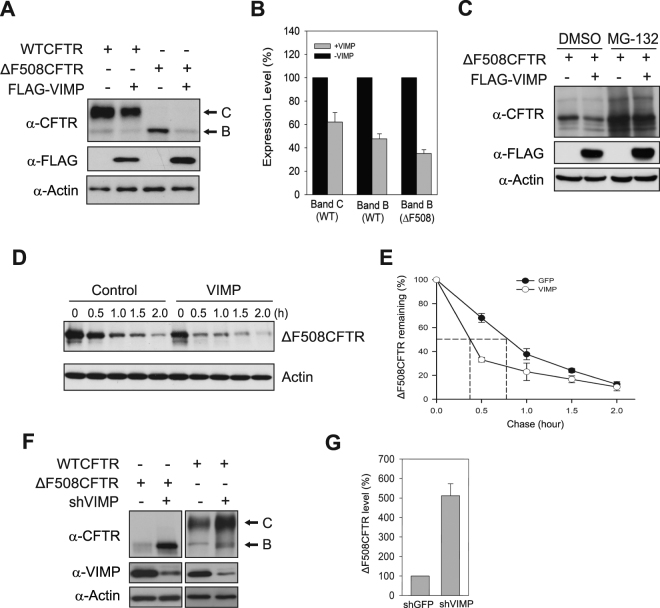
VIMP facilitates the degradation of CFTRΔF508. (**A**) Overexpression of VIMP enhances the degradation of CFTRΔF508 and WT CFTR. Plasmid encoding VIMP was co-transfected with vectors expressing WT CFTR or CFTRΔF508 into HEK293 cells. The steady-state levels of WT CFTR, CFTRΔF508, VIMP and actin were measure by immunoblotting analysis. (**B**) Quantitation of the steady-state levels of immature (band **B**) and mature (band **C**) forms of WT CFTR and CFTRΔF508 from (**A**). Data presented were from three independent experiments and shown as the mean ± SD. (**C**) Degradation of CFTRΔF508 is mediated by the proteasome. HEK293 cells expressing CFTRΔF508 with or without overexpression of VIMP were treated with MG132 (10 μM) or DMSO for 4 h. Cells were harvested and subjected to immunoblotting analysis. (**D**) CHX chase experiment was carried out to measure the turnover kinetics of CFTRΔF508 with or without VIMP overexpression. (**E**) Decay curves of CFTRΔF508 in the presence and absence of VIMP overexpression. Data presented were from three independent CHX-chase experiments as (**D**) and shown as the mean ± SD. (**F**) CFTRΔF508 and WT CFTR were stabilized by knockdown of VIMP. shVIMP or a scramble vector was co-transfected with CFTRΔF508 or WT CFTR plasmids into HEK293 cells. The protein expression levels were evaluated by immunoblotting analysis. (**G**) Quantitation of the blots in (**F**). Data shown here are the mean ± SD from three independent experiments.

The binding of VIMP to CFTRΔF508 at ER membrane dilations prompted us to examine the effect of VIMP on the degradation of immature CFTRΔF508. We co-expressed VIMP with CFTRΔF508 in HEK293 cells. Immunoblotting analysis showed that overexpression of VIMP reduced the steady-state level of immature CFTRΔF508 (“band B”) by 60% (Fig. 2A,B). VIMP-induced degradation of CFTRΔF508 was mediated by the proteasome because the proteasome inhibitor MG132 markedly recovered the steady-state level of CFTRΔF508 (Fig. 2C). We further evaluated the effect of VIMP overexpression on the turnover kinetics of CFTRΔF508 using the cycloheximide (CHX)-chase assay. As shown in Fig. 2D,E, overexpression of VIMP significantly shortened the half-life of CFTRΔF508 from ~50 min to ~25 min. To examine the role of endogenous VIMP in the degradation of CFTRΔF508, we used RNA interference to reduce VIMP expression. Specifically, a VIMP-targeting shRNA vector was transfected into CFBE41o^−^ cells expressing CFTRΔF508. Immunoblotting analysis showed that the steady-state level of CFTRΔF508 was substantially increased by knockdown of VIMP (Fig. 2F, left panels). Quantitation of the CFTRΔF508 blots by ImageJ revealed that its steady-state level increased ~5 fold upon knockdown VIMP (Fig. 2G). Thus, VIMP facilitates the degradation of immature CFTRΔF508. VIMP also enhanced the degradation of wildtype (WT) CFTR. The steady-levels of immature (“band B”) and mature (“band C”) forms of WT CFTR decreased by 50% and 30%, respectively, when VIMP was overexpressed (Fig. 2A,B). On the other hand, knockdown of VIMP expression increased the expression level of WT CFTR (Fig. 2F).

**Figure 3:**
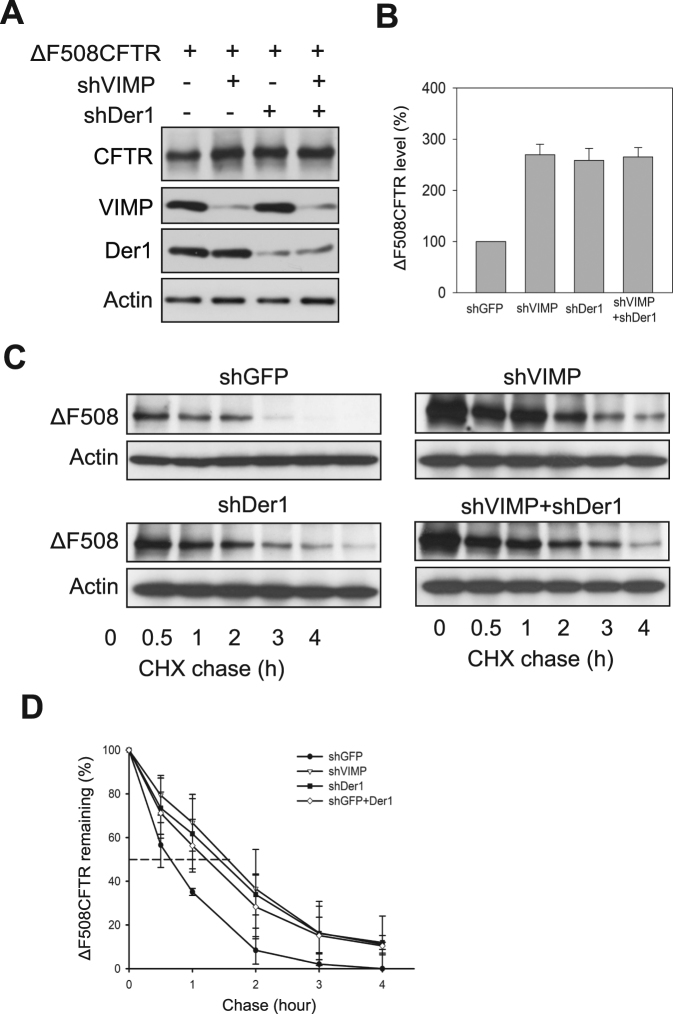
VIMP and Derlin-1 act in the same pathway for CFTRΔF508 degradation. (**A**) Immunoblotting analysis for the steady-state level of CFTRΔF508 with knockdown of VIMP or Derlin-1 or both. (**B**) Quantitation of the data of (**A**). Data shown are from three independent experiments and presented as the mean ± SD. (**C**) CHX chase assay was employed to measure the turnover rate of CFTRΔF508 with knockdown of VIMP or Derlin-1 or both. (**D**) Decay curves of CFTRΔF508. The decay curves were prepared from three independent experiments and shown as the mean ± SD.

Our previous study showed that Derlin-1 plays a key role in mediating CFTRΔF508 degradation^17^. To evaluate if VIMP and Derlin-1 act in the same pathway for CFTRΔF508 degradation, we performed a double knockdown experiment. shRNA vectors targeting VIMP and Derlin-1 were co-transfected with CFTRΔF508 into HEK293 cells, controlled by single knockdown of VIMP or Derlin-1. We found that the increase of CFTRΔF508 protein level by knockdown of both VIMP and Derlin-1 was comparable to that caused by single knockdown of VIMP or Derlin-1 (Fig. 3A,B). CHX-chase analysis showed that knockdown of VIMP or Derlin-1 or both similarly extended the half-life of CFTRΔF508 to ~80 min from 50 min (Fig. 3C,D). These results demonstrated that VIMP and Derlin-1 are involved in same degradation pathway eliminating CFTRΔF508.

### VIMP is required for RNF5-mediated degradation of CFTRΔF508

**Figure 4:**
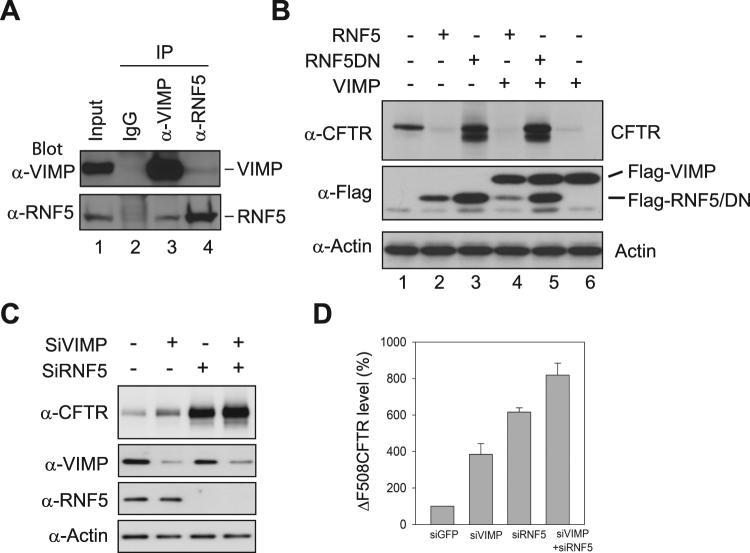
VIMP is required for RNF5-mediated degradation of CFTRΔF508. (**A**) VIMP interacts with RNF5. Cell extracts were subjected to IP with pre-immune IgG (lane 2), anti-VIMP (lane 3), and anti-RNF5 (lane 4) antibodies, followed by immunoblotting with anti-VIMP (upper panel) and anti-RNF5 (lower panel) antibodies. (**B**) Overexpression of RNF5 and VIMP facilitates the degradation of CFTRΔF508. RNF5 or its dominant negative mutant RNF5DN was co-transfected with VIMP (lanes 4–6) or a control vector (lanes 1–3) into HEK293 cells expressing CFTRΔF508. The expression levels of CFTRΔF508, RNF5, RNF5DN, and VIMP were measured by immunoblotting analysis. (**C**) Knockdown of RNF5 stabilizes CFTRΔF508. siRNA oligos targeting RNF5 and VIMP were transfected individually or in combination into CFBE41o^−^ cells expressing CFTRΔF508. Immunoblotting analysis was performed to compare the steady-state level of CFTRΔF508. (**D**) Quantitation of the blots of CFTRΔF508 in (**C**). Data shown were presented as the mean ± SD from three experiments.

An early report showed that two E3 ubiquitin ligase complexes drive the degradation of CFTRΔF508 sequentially^19^. One complex is associated with the ER membrane, consisting of the E3 RNF5 (also known as RMA1), the E2 Ubc6e, and Derlin-1, while the other complex containing the CHIP E3 and Hsc70 is present in the cytosol. The RNF5 E3 detects folding defects of CFTRΔF508 concomitant with its synthesis on ER-attached ribosomes, whereas the cytosolic CHIP E3 acts posttranslationally to monitor the folding status of CFTRΔF508. It remains unknown whether VIMP is required for the degradation of CFTRΔF508 driven by RNF5 and CHIP. To address this question, we first examined if these two E3s interact with VIMP. Specifically, we applied reciprocal co-IP experiments to measure the interaction between endogenous VIMP and RNF5 or CHIP. We found that RNF5 was co-precipitated with VIMP by an anti-VIMP antibody but not a control IgG (Fig. 4A, lanes 2, 3). Likewise, VIMP was co-precipitated with RNF5 by an anti-RNF5 antibody (lane 4). However, co-IP experiments did not detect interaction between VIMP and CHIP (data not shown). We then assessed the cooperation of VIMP and RNF5 in ERAD of CFTRΔF508. VIMP or a control vector was co-overexpressed with RNF5 or RNF5DN, a dominant negative mutant with a C42S substitution in the RING domain, in HEK293 cells expressing CFTRΔF508. Overexpression of RNF5 enhanced the degradation of CFTRΔF508, whereas RNF5DN markedly stabilized CFTRΔF508 (Fig. 4B, lanes 1–3). Interestingly, overexpression of RNF5DN significantly counteracted the effect of VIMP on CFTRΔF508 degradation (Fig. 4B, compare lanes 5 and 6). We next measured the effects of knockdown of RNF5 and/or VIMP on CFTRΔF508 degradation. Small interfering RNA oligos targeting RNF5 and VIMP were transfected individually or in combination into CFBE41o^−^ cells expressing CFTRΔF508. Immunoblotting analysis showed that the steady-state level of CFTRΔF508 was substantially increased by the knockdown of RNF5 (Fig. 4C, compare lanes 1 and 3). Double knockdown of RNF5 and VIMP further increased the stability of CFTRΔF508 (Fig. 4C,D). Together, these results demonstrate that VIMP plays an important role in RNF5-mediated degradation of CFTRΔF508.

### Rescue of the chloride channel function of CFTRΔF508 by downregulation of VIMP and VX809 treatment

**Figure 5:**
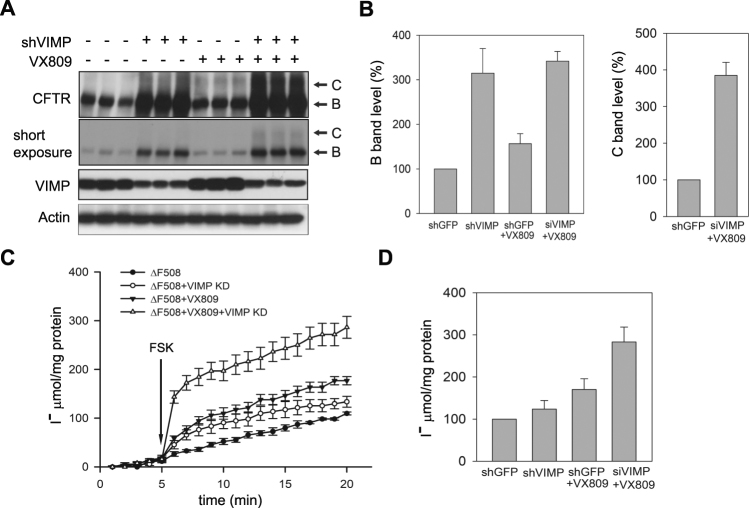
Rescue of the chloride channel function of CFTRΔF508 by VX809 and knockdown of VIMP. (**A**) Increase of mature CFTRΔF508 by combined treatment of VX809 and knockdown of VIMP. HEK293 cells were transfected with siVIMP or a scramble vector, followed by treatment with or without VX809. The steady-state levels of immature (**B** band) and mature (**C** band) CFTRΔF508 were detected by immunoblotting analysis. Experiments were performed in triplicate. (**B**) Quantitation of the B and C bands of CFTRΔF508 in (**A**). Data shown were presented as the mean ± SD. (**C**) Iodide efflux analysis for the channel activity of CFTRΔF508. Transfected HEK293 cells as in (**A**) were applied to I^−^ efflux experiments described previously^17^. The time course of forskolin-stimulated CFTR channel-mediated I^−^ efflux was determined. Each point represents the mean ± SD of 3 to 5 experiments. The time of forskolin (Fsk) addition is indicated by the arrow. (**D**) Comparison of the maximal CFTRΔF508 channel signals in different HEK293 transfectants. The stimulation of I^−^ efflux by forskolin reached a peak at approximately 15 min after drug application. Data shown were presented as the mean ± SD from 3–5 experiments.

While depletion of VIMP helps to prevent CFTRΔF508 from degradation, it is not sufficient for CFTRΔF508 to reach the plasma membrane to exhibit its chloride channel function. We wanted to test if reducing the VIMP expression level can enhance the effect of CF corrector VX809, a drug that allows a fraction of CFTRΔF508 to fold properly and mobilize from ER to cell surface for normal functioning. HEK293 cells were transfected with siVIMP, followed by treatment with or without VX809. Although knockdown of VIMP markedly increased the steady-state level of immature CFTRΔF508, mature CFTRΔF508 was hardly detected (Fig. 5A). However, the treatment of VX809 following VIMP knockdown yielded a substantial fraction of mature CFTRΔF508 (Fig. 5A,B). We also examined the ion channel activity of CFTRΔF508 upon VIMP knockdown and VX809 treatment using iodide efflux experiment^20^. VX809 increased the channel activity of CFTRΔF508 by ∼80%, whereas knockdown of VIMP only had a marginal effect (Fig. 5C,D). Remarkably, the transport capacity of CFTRΔF508 was dramatically increased (2.8 fold) by VX809 when VIMP expression was also suppressed (Fig. 5C,D). These data demonstrate that lowing VIMP expression can synergize the effect of VX809 through increasing the steady-state level of CFTRΔF508 by suppressing ERAD.

## Discussion

ERAD is a complicated degradation pathway including multiple steps: substrate recognition, retrotranslocation, ubiquitylation, and degradation. CFTR is a well known substrate of ERAD^21,22^. Although extensive studies have been conducted on CFTR degradation, some details are still missing. Our laboratory previously showed that Derlin-1 preferentially mediates CFTRΔF508 degradation^17^. Derlin-1 is considered as the central molecule that transports misfolded substrates from ER lumen to cytosol during ERAD, and the substrates are then submitted to VCP complex that is recruited to Derlin-1 by VIMP^3–5,23^. It has been unclear whether and how VIMP contributes to the degradation of CFTRΔF508. In this study, we demonstrate that VIMP is required for the degradation of CFTRΔF508. Using RNA interference, we found that knockdown of VIMP or Derlin-1 or both exhibited a similar effect on the degradation of CFTRΔF508, indicating that VIMP and Derlin-1 act in the same pathway. This is in line with the physical interaction between VIMP and Derlin-1. Moreover, we demonstrated that VIMP interacts with CFTRΔF508 and RNF5. These observations suggest that, in addition to being a linker between Derlin-1 and VCP, VIMP may participate in recruiting substrate and E3 in ERAD of CFTRΔF508.

In addition to RNF5 and CHIP, several other E3s have been implicated in CFTRΔF508 degradation, including gp78, Hrd1, Nedd4-2, and RNF185^24–28^. Among these E3s, RNF5 is likely the major one targeting CFTRΔF508 for degradation. Our previous study showed that 70–80% of CFTRΔF508 is degraded cotranslationally^17^. RNF5 is responsible for cotranslational degradation of CFTRΔF508, whereas CHIP acts posttranslationally^19^. gp78 appears to function as an E4 downstream of RNF5^25^. Hrd1 does not directly target CFTRΔF508. Instead, it inhibits CFTRΔF508 degradation by acting as an E3 for gp78^24^. The role of Nedd4–2 in CFTRΔF508 degradation is inconclusive. While one report suggests that Nedd4–2 facilitates CFTRΔF508 degradation, the other study indicates no such effect^26,27^. RNF185 is homologous to RNF5 and, as RNF5, directs CFTRΔF508 for cotranslational degradation^28^. Surprisingly, however, simultaneous deletion of RNF5 and RNF185 drastically inhibits CFTRΔF508 degradation not only cotranslationally but also posttranslationally. It will be interesting to explore the functional interaction between RNF5 and RNF185 in controlling CFTRΔF508 degradation. It also remains to be determined if VIMP is required for RNF185-mediated degradation of CFTRΔF508.

Our data indicate that VIMP utilizes RNF5 as the E3 ligase to direct the bulk of CFTRΔF508 for proteasomal degradation. Simultaneous overexpression of VIMP and RNF5 promoted more CFTRΔF508 degradation than separate overexpression of VIMP or RNF5. Consistently, double knockdown of VIMP and RNF5 stabilizes CFTRΔF508 to a greater extent than single knockdown. An early work suggested that RNF5 and CHIP could regulate CFTRΔF508 degradation at different stages^19^. RNF5 targets misfolded CFTR at early stage during biosynthesis, whereas CHIP recognizes CFTR after its translation. Our finding that VIMP directly binds CFTRΔF508 and RNF5 indicates that VIMP is involved in early degradation of CFTR. In fact, the majority of CFTRΔF508 is degraded cotranslationally^17^. Of note, VIMP may contribute to ERAD in different aspects as well. It has been reported that VIMP regulates rough ER shape, which may affect the distribution of retro-translocon proteins^29^. It is also possible that VIMP (a selenocysteine-containing protein) acts as a reductase to help form disulfides in substrates and facilitate their retro-translocation from ER to cytosol^30^.

One interesting result from the current study is that knockdown of VIMP significantly enhances the rescue effect of CF corrector VX809. The combination of VX809 and VIMP knockdown generates much higher CFTR chloride channel activity than VX809 treatment alone. This increase results from the stabilization of immature CFTRΔF508 by downregulation of VIMP expression and VX809-assisted folding, which leads to 3.5-fold increase of mature CFTR expression. Until now, no effective medicine for CFTRΔF508-associated CF patients has been found^31^. The strategies for CF medicine discovery are typical one-step rules, which attempt to find a chemical as a corrector for mutated CFTR or as an activator or inhibitor to increase or decrease CFTR-associated protein expression^12,32–34^. Here we propose a two-step rule: prevent CFTRΔF508 from ERAD, and then help the rescued CFTRΔF508 to fold and migrate to cell membrane functioning as a chloride channel. This study presents VIMP as a potential target for blocking early degradation of CFTRΔF508, which may be crucial in the development of efficient medicines for the treatment of CF patients.

### Experimental procedures

#### General materials

Mouse monoclonal anti-CFTR antibody was obtained from University of North Carolina. Rabbit polyclonal anti-VIMP and anti-RNF5 were purchased from Abcam. The antibodies for FLAG epitope, Myc, Derlin-1, GFP, actin were purchased from Sigma and Cell Signaling. Normal rabbit IgG, HRP-labeled goat anti-mouse IgG, and HRP-labeled goat anti-rabbit IgG were purchased from Abcam. The vectors encoding Flag-VIMP, pEGFP, Derlin-1, RNF5, RNF5DN, shVIMP, shDer1, shGFP, WT CFTR and CFTRΔF508 were described previous^17^. All siRNA oligos were purchased from Dharmacon.

#### Cell culture and transfection

HEK 293 cells were cultured in Dulbecco’s modified Eagle’s medium (Invitrogen) supplemented with 10% fetal bovine serum. CFBE41o^−^ parental cells were cultured in MEM with 10% fetal bovine serum, and CFBE41o-∆F508 cells were grown in the same medium as CFBE41o^-^ parental cells except for addition of 0.5 μg/L puromycin. All cells were maintained in a humidified atmosphere containing 5% CO_2_ at 37 °C. Cells transfections were performed using Lipofectamine 2000 (Invitrogen) for plasmid vectors and Lipifectamine RNAiMAX (Invitrogen) for siRNA oligos, following the manufacturer’s instruction. Cells were harvested 48 h after transfection.

#### Immunoprecipitation and immunoblotting

Cells were lysed by sonication in lysis buffer (50 mM HEPES, 150 mM NaCl, 1 mM EDTA, 1% NP40, 10% glycerol and protease inhibitors cocktail). Pre-cleared cell lysates (~500 μg of each) were mixed with appropriate primary antibodies at 4 °C for 1.5 h. Protein A- or G-coupled Dynabeads (25 μl) were then added and incubated at room temperature for 1 h with gentle rotation. Immunocomplexes were washed with lysis buffer four times and resuspended in SDS sample buffer and subjected to immunoblotting analysis. Specifically, protein samples were resolved by SDS-PAGE and transferred to PVDF membranes, which were blocked at room temperature for 1 h with 5% (w/v) skim milk powder in TBST (10 mM Tris (pH 8.0), 150 mM NaCl, 0.05% Tween 20). The blots were incubated with primary antibodies at room temperature for 1 h. The blots were then washed four times with TBST and incubated with 2 μg/ml horseradish peroxidase-conjugated secondary antibodies (Sigma) in TBST with 10% fetal bovine serum for 1 h, followed by five washes with TBST. The reactive bands were visualized by incubation with enhanced chemiluminescence substrates (PerkinElmer Products) and exposure to X-ray film (Eastman Kodak Co).

#### Production of VIMP and CFTRΔF508 by the TNT T7 coupled reticulocyte lysate system

Each reaction contained 50 μL TNT T7 coupled reticulocyte lysate (Promega) with DNA templates pCDNA3.1-ΔF508CFTR or pCDNA3.1-ΔF508CFTR plus pCDNA3.1-Flag-VIMP. Reactions were set up at 30 °C for 90 minutes. Three microliter reaction mix was taken out as input control, whereas the remaining was diluted with cell lysis buffer containing protease inhibitors to 500 μL and subjected to IP with anti-CFTR antibody and Protein G Dynabeads at room temperature for 1 h. The immunoprecipitates were washed four times with cell lysis buffer, followed by SDS-PAGE (12.5% gel) and immune blotting with anti-VIMP and anti-CFTR antibodies.

#### Cycloheximide (CHX) chase analysis

Forty-eight hours after transfection, cells were digested with trypsin and continued cultured in fresh medium supplemented with CHX (20 μg/ml). Equivalent volume of cell suspensions was harvested at different time points. Cell extracts were subjected immunoblotting analysis with appropriate antibodies.

#### Immunofluorescence and confocal microscopy

CFBE41o^−^-CFTRΔF508 cells were fixed in 2% paraformaldehyde and permeabilized with 2% paraformaldehyde plus 0.1% Triton X-100, then washed three times with buffer B (phosphate-buffered saline with 0.5% bovine serum albumin and 0.15% glycine, pH 7.4). After blocking with 20% goat serum, cells were incubated with the appropriate primary antibodies for 1 h, followed by three washes with buffer B and subsequent incubation with Alexa 488- (green) or Alexa568 (red)-labeled secondary antibodies (Molecular Probes) for 1 h. After washing with buffer B, the coverslips were mounted for confocal microscopy.

#### Iodide Efflux experiments

HEK293 cells were transfected with CFTRΔF508 plasmid and shVIMP or a scramble vector. On next day, cells were incubated with10 μmol/L VX809 for 24 h before subjected to iodide efflux assay as previously described^20^. Specifically, cells were washed with 2 ml warmed I^−^-loading buffer (136 mM sodium iodide, 3 mM potassium nitrate, 2 mM calcium nitrate, 11 mM glucose and 20 mM HEPES, pH 7.4 with NaOH) twice, and then incubated with 2 ml I^−^-loading buffer at 37 °C and 5% CO_2_ for 45 min. Cells were gently rinsed twice with 4 ml warmed I^−^-free efflux buffer (136 mM sodium nitrate, 3 mM potassium nitrate, 2 mM calcium nitrate, 11 mM glucose and 20 mM HEPES, pH 7.4). Fresh warmed I^−^-free efflux buffer was slowly dropped on the cell dishes that were placed on 37 °C warm bath plate. I^−^-selective electrode (Orion model 94–53) and a reference electrode (Orion model 90-01) were connected to a pH meter (Accumet 915). After incubated with electrodes for 5 min, 1 μmol /L forskolin was added in the cells, and the numbers on pH meter were recorded every 1 min.

#### Statistical analyses

Statistical analyses were performed by Student’s test using statistical software. All quantitative data presented in this study were from 3–5 independent experiments and shown as the mean ± SD.

## Electronic supplementary material

Supplementary Information
