# Supplementary material for: Dissection of the Role of VIMP in Endoplasmic Reticulum-Associated Degradation of CFTRΔF508
Source: Sci Rep. 2018 Mar 19;8:4764. doi: 10.1038/s41598-018-23284-8 (PMC5859151; doi:10.1038/s41598-018-23284-8)
Supplement: Supplementary file 1 — Supplementary Information [file 41598_2018_23284_MOESM1_ESM.pdf]

Supplementary Information

Dissection of the Role of VIMP in Endoplasmic Reticulum-Associated Degradation of CFTRΔF508

Xia Hou[1, 2], Hongguang Wei[1], Carthic Rajagopalan[1], Hong Jiang[1], Qingtian Wu[2], Khalequz Zaman[3], Youming Xie[4,*], Fei Sun[1,*]

[1]Department of Physiology, Wayne State University School of Medicine, Detroit, MI 48201, USA. [2]Department of Biochemistry and Molecular Biology, Jiamusi University School of Basic Medicine, Jiamusi, Heilongjiang, 154007, China. [3]Department of Pediatrics, Case Western Reserve University, Cleveland, OH 44106. [4]Karmanos Cancer Institute and Department of Oncology, Wayne State University School of Medicine, Detroit, MI 48201, USA.

*Correspondence to Fei Sun (fsun@med.wayne.edu) or Youming Xie (xiey@karmanos.org)

Tel: 313-577-2428, Fax: 313-577-5494

A

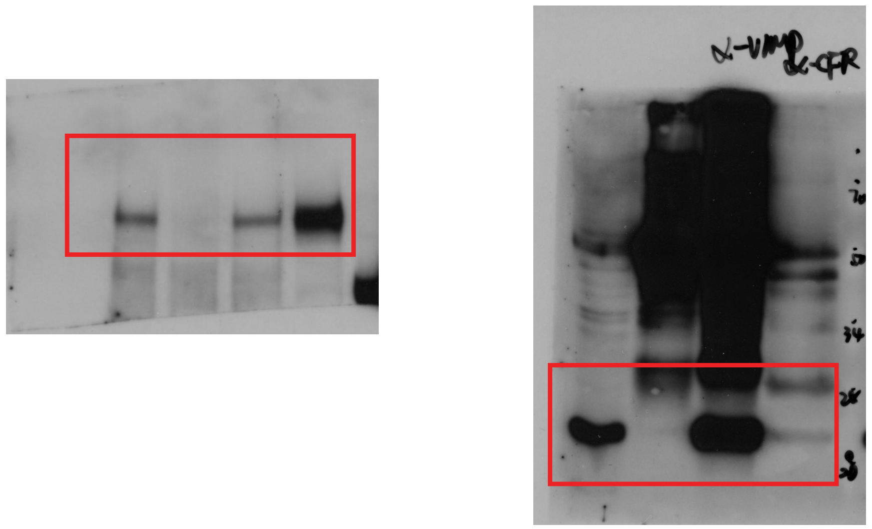

B

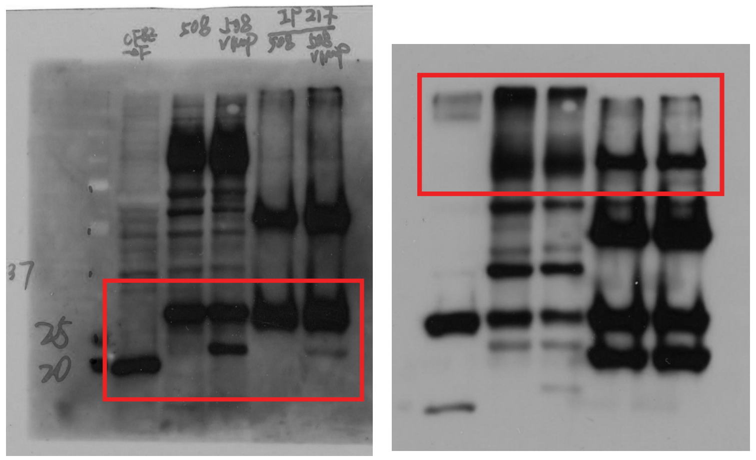

Figure S1. Full images used for generation of Figs 1A, B in main text.

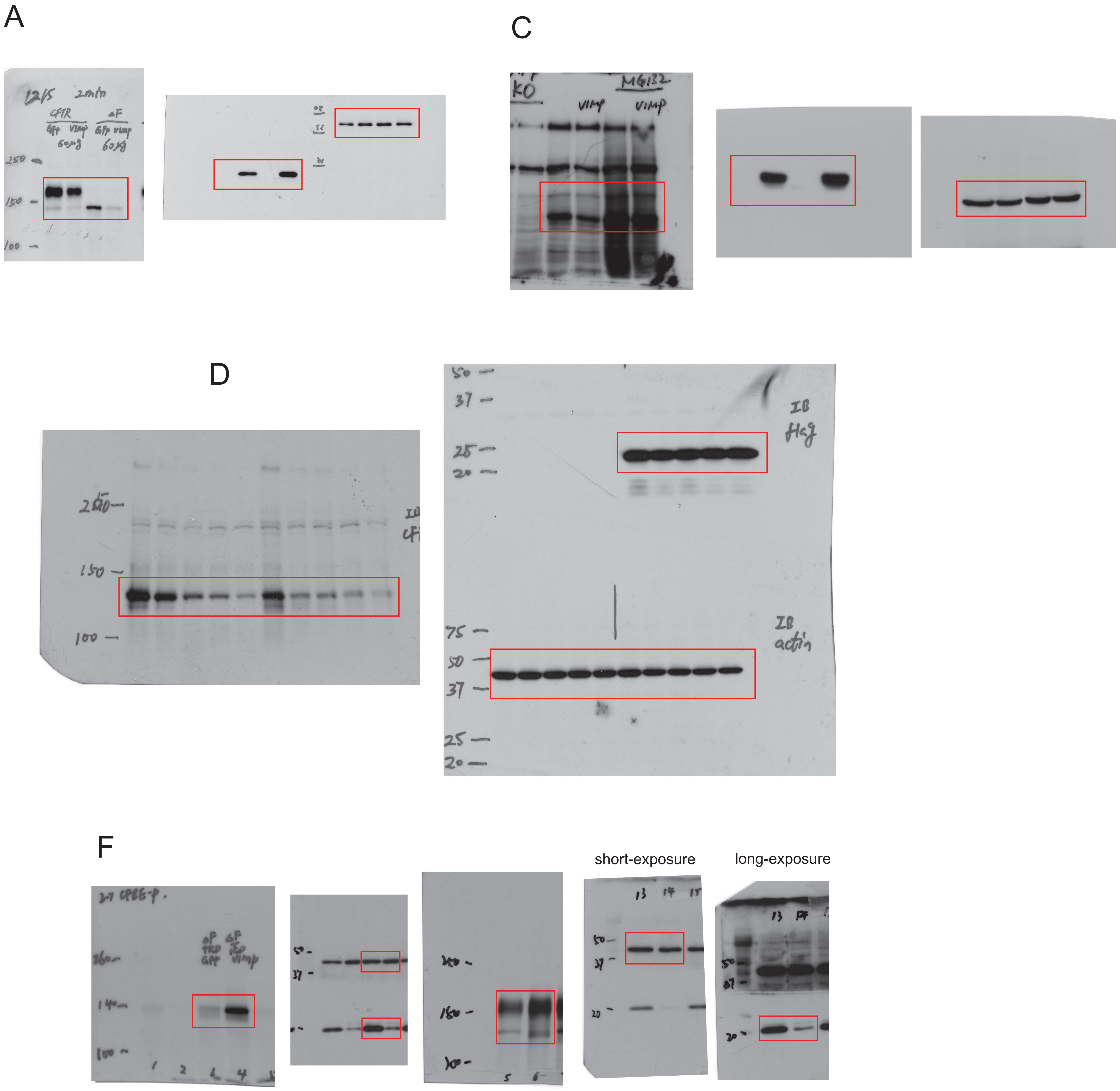

Figure S2. Full images used for generation of Figs 2A, C, D, F in main text.

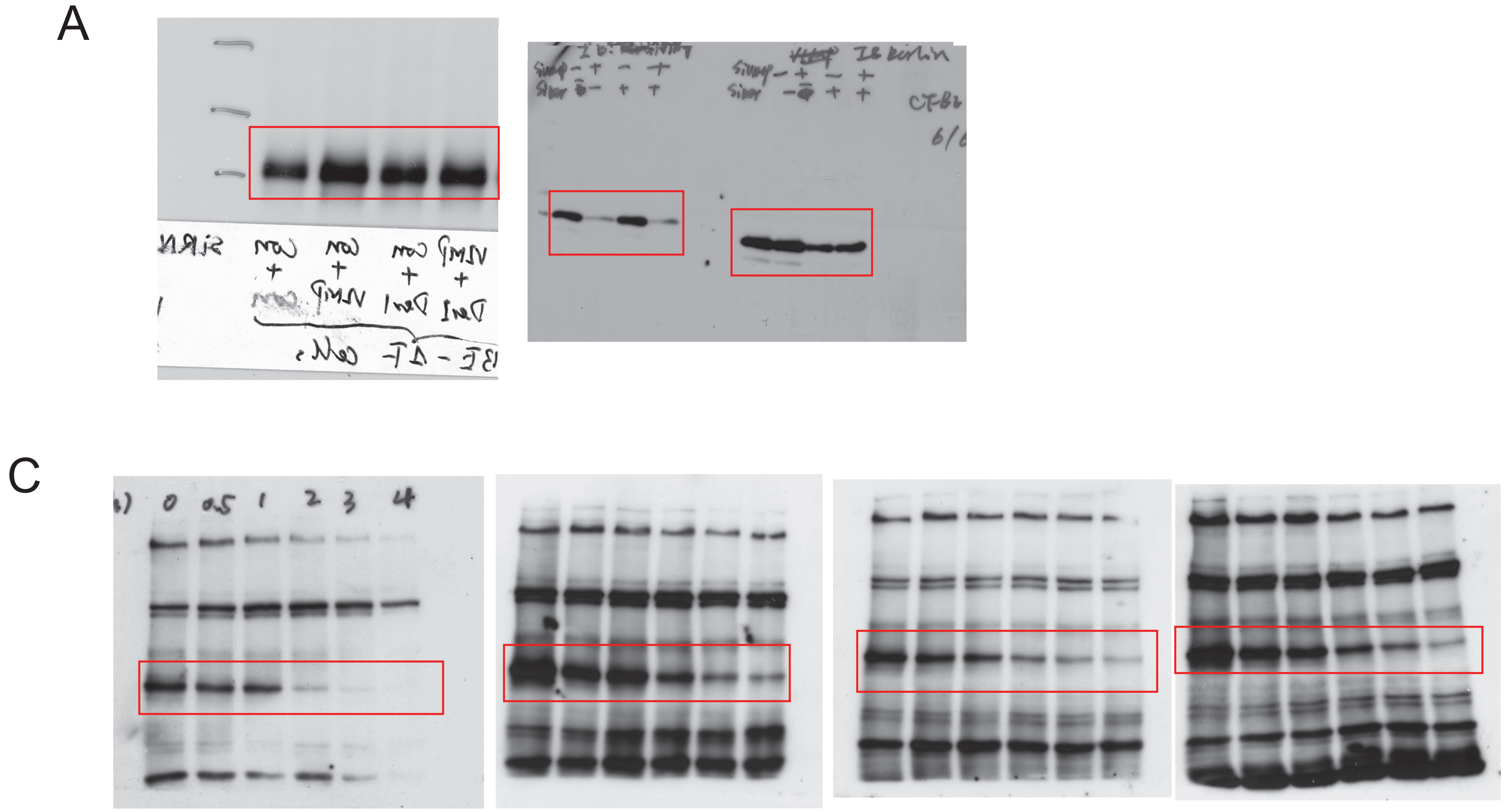

Figure S3. Full images used for generation of Figs 3A, C in main text.

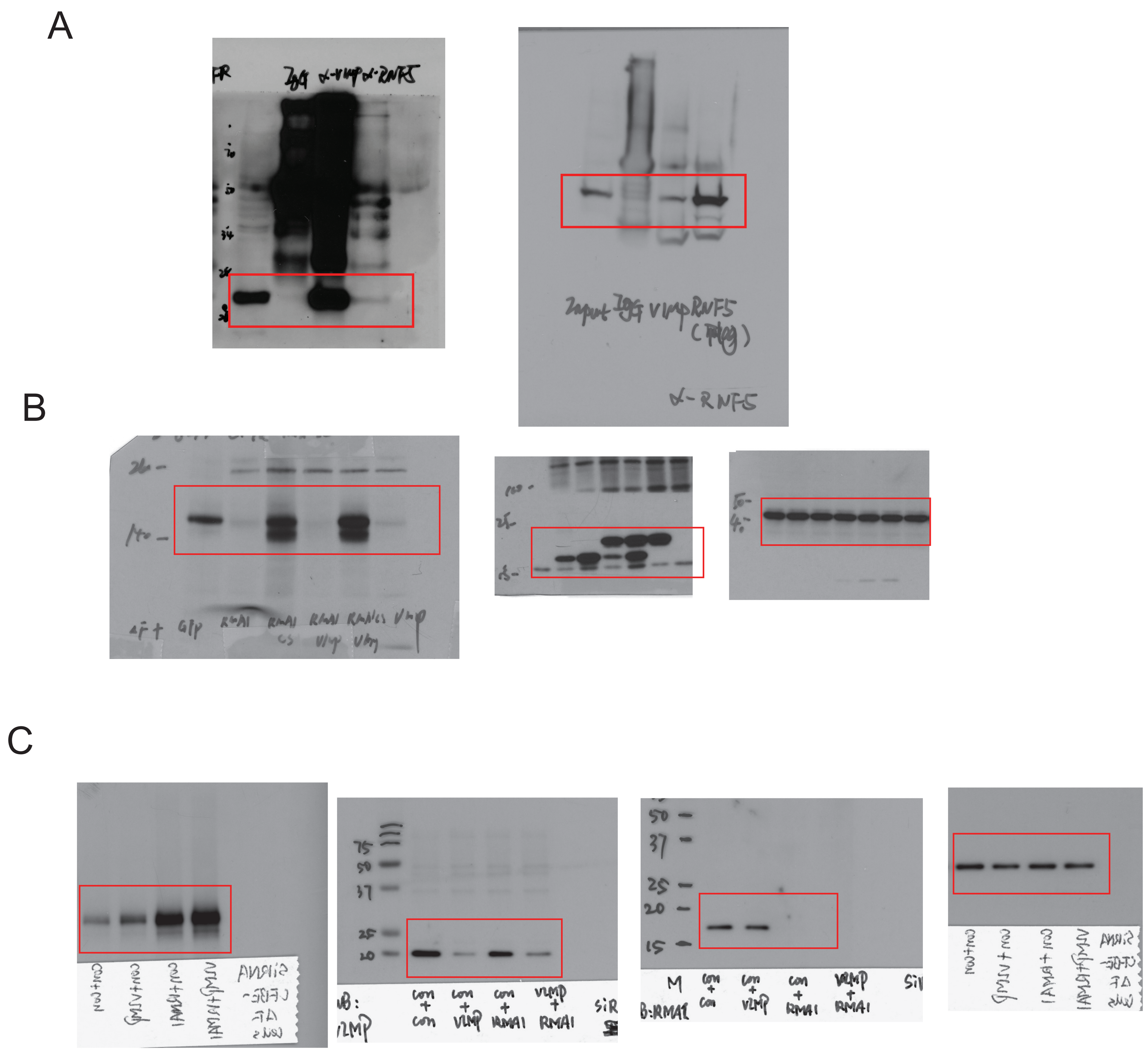

Figure S4. Full images used for generation of Figs 4A, B, C, D in main text.

A

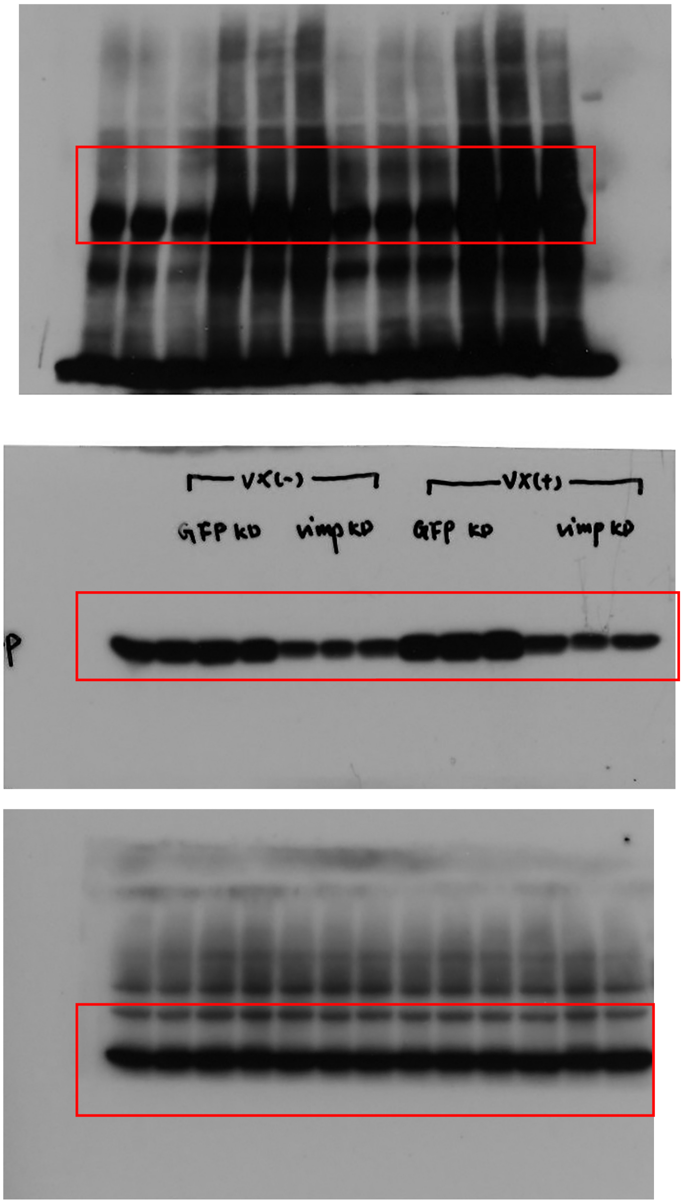

Figure S5. Full images used for generation of Fig 5A in main text.
